# Evaluation of IRF7 mRNA and its Association with Promoter Methylation in Kashmiri (North-Indian) Patients with Systemic Sclerosis: A Case-Control Study

**DOI:** 10.31138/mjr.040124.eoi

**Published:** 2024-12-31

**Authors:** Sakeena Ayub, Zafar A Shah, Fayaz A Sofi, Roohi Rasool, Tabasum Shafi, Mushtaq Dangroo, Muzaffar Bindroo, Imtiyaz A. Bhat

**Affiliations:** 1 Department of Immunology & Molecular Medicine;; 2 Division of Rheumatology, Department of Internal Medicine, SKIMS, Srinagar, India

**Keywords:** systemic sclerosis, IRF-7, DNA methylation, gene expression

## Abstract

**Introduction:**

The interferon regulatory factor 7 (IRF7), a member of the IRF family of transcription factors, plays a major role in the regulation of numerous aspects of an immune response and has increasingly been surveyed to determine the aetiology and pathogenesis of systemic sclerosis (SSc). Objective: This study aimed to investigate the transcriptional levels of IRF7 mRNA in peripheral blood mononuclear cells (PBMCs) and the impact of promoter methylation on IRF7 mRNA expression in SSc patients compared to healthy controls.

**Methods:**

PBMCs were obtained from confirmed 40 naïve SSc cases and 20 healthy controls for IRF-7 expression and methylation analysis. mRNA expression was performed using the quantitative real-time polymerase chain reaction (SYBR green method) concerning the housekeeping gene. A promoter methylation profile study was carried out by bisulfite treatment of DNA, followed by methylation-specific polymerase chain reaction (MS-PCR) in SSc cases against controls.

**Results:**

The relative expression analysis revealed that the selected IRF7 gene was upregulated in the patient group compared to healthy controls (p=0.003). In addition, mRNA expression of IRF7 was significantly increased in the limited cutaneous group compared to the diffuse cutaneous group. Moreover, SSc cases had hypomethylated IRF7 promoters compared to controls, and the significant impact of IRF7 promoter methylation on mRNA expression was observed (p=0.001).

**Conclusion:**

IRF7 overexpression in PBMCs from SSc patients may be caused by IRF7 promoter demethylation, and this aberrant expression of IRF7 in SSc might provide a link between the prominent IFN signature and the development of SSc.

## INTRODUCTION

Systemic sclerosis (SSc) is an uncommon autoimmune disease is marked by fibrotic, immunological, and vascular alterations that commonly impact the skin and internal organ systems. Patients are typically divided into categories with diffuse or limited SSc disease. In contrast to limited instances, diffuse cases often advance the disease more quickly, thicken the skin more widely, impact the internal organ systems faster, and have a worse prognosis. In addition to having high morbidity and death rates, SSc mostly affects young women and frequently manifests during the reproductive years. It also substantially influences many facets of quality of life.^1^

The aetiology of SSc is highly variable and complicated, but it appears that genetics and particular environmental factors interact to control the development and course of the disease.^2^ Exposure to chemical substances (such as silica or organic solvents) or infectious pathogens has long been linked to a higher risk of contracting a disease. Recent research suggests that genetics and epigenetics may play important roles in developing SSc.^3^ This new wave of studies is increasingly showing abnormal epigenetic modifications in genes relevant to the pathogenesis of SSc.^4^ A gene’s transcription will be impeded if its promoter region is sufficiently methylated because fewer transcription factors will be able to bind to it. On the other hand, DNA transcription is activated by low methylation of the promoter.^5^ According to several studies,^6–9^ type I IFNs have been linked to the pathogenesis of systemic autoimmune diseases such as Sjögren’s syndrome, SLE, and SSc. As a result, it is probable that a type I IFN signature also has a particular role in the pathophysiology of SSc. Unfortunately, there are presently no effective treatments available for the fibrotic side effects of SSc, and the mortality rate from the condition is still quite high. These findings prompted us to conduct a study investigating the role of IRF-7 promoter methylation and mRNA expression in the aetiology of SSc.

## METHODS

### Subject recruitment and ethics

A case-control association study was designed to assess the role of the IRF7 gene in systemic sclerosis susceptibility. At enrolment, patients were diagnosed with Scleroderma and profiled into the DcSSc and LcSSc subgroups using the criteria proposed by LeRoy et al. and updated criteria proposed by the European League Against Rheumatism.^10^ SSc was defined according to Internationally Agreed Guidelines. A total of 40 naïve cases of SSc with no prior treatment who visited this tertiary care centre during our study period were enrolled in our study. The study included 20 age and sex-matched healthy volunteers with no history of inflammatory or autoimmune disorders as controls. Patients with overlapping Sjögren’s syndrome, myositis, rheumatoid arthritis, or systemic lupus were excluded from the study. Disease duration was measured in years from the commencement of the first non-Raynaud’s phenomenon symptom to the patient’s first and second investigations throughout the study. All of the patients were given an information pamphlet and were asked to provide written informed permission on a special consent form. The patient’s examination findings were utilised to collect information on the anti-topo I, anti-centromere, and anti-nuclear autoantibody profiles of SSc patients.

### Sample Collection

A total of 3ml of peripheral blood sample was collected in EDTA vials (200μl of 0.5M, pH=8.0) from both Scleroderma and healthy controls of which 1ml was stored at −80 for DNA extraction until further processing and the remaining 2ml was processed immediately for RNA extraction.

### Methylation-specific PCR (MS-PCR)

Genomic DNA was extracted from peripheral blood following standard procedures. Extracted DNA’s quality and its concentration were checked on 1% Agarose gel electrophoresis and spectrophotometer (Nanodrop Eppendorf AG, Hamburg, Germany). The bisulfite alteration of the 1.5–2ug sample DNA was carried out according to the protocol given by the commercially available bisulfite modification kit (EZ DNA Methylation-Gold Kit, Cat. No: D5005, ZYMO Research, Orange, CA) and subjected to PCR for methylated and modified unmethylated DNA using specific primers. With the assistance of software programs for CpG islands identification and primer design, the primer pairs for the methylation-specific PCR (**Table 3**) in this study were designed according to the same principle. The bisulfite-modified genomic DNA was amplified by MS- PCR reactions in a total volume of 25μl containing 10X PCR buffer, 1.25mM MgCl_2_, 200mM dNTPs (Invitrogen, Germany), 2μl template DNA, 1U Taq polymerase and 20pmol of primers specified for either methylated or the modified unmethylated DNA. The PCR thermocycler conditions were as follows: initial denaturation at 95°C for 5 min, followed by 35 cycles of 94°C for 35 sec, X°C for 35 sec, and 72°C for 35 sec, with the final extension of 72°C for 10 min. The PCR reaction was carried out in two separate tubes specific for methylated or unmethylated sequences. To verify the PCR results, representative bands from each target and housekeeping gene Human methylated DNA control and Human non-methylated control (ZYMO Research) were gel-purified on a 2% agarose gel yielding a band of 196bp for methylated and 208bp for an unmethylated product and visualised under UV illumination.

**Table 3.** Primer sequences for MS-PCR.

**Gene**	**Gene Bank No**	**Sense 5’-3’**	**Antisense 5’-3’**	**Genomic position**	**Size (bp)**
**IRF-7 (M)**	NT_008953.8	GTTTCGCGGAGTTGAGAATC	TATAACCGACGCGCACAC	+161? to +356	196
**IRF-7 (U)**	NT_008953.8	GGTGGGGTTTTGTGGAGT	TACAAATATAACCAACACACACAC	+155 to +362	208

### RNA Extraction and cDNA synthesis

The blood samples for RNA were processed immediately after collection. PBMCs were separated by using Ficoll-hypaque mediated (HIMEDIA HiSep^TM^ LSM 1077) density gradient centrifugation. RNA was extracted from isolated cells by using the Trizol-Chloroform method. A Microvolume UV-Vis Spectrophotometer (Nanodrop Eppendorf AG, Hamburg, Germany) was used to measure the concentration of the acquired RNA at a wavelength of 260 nm. On a 1.2% agarose gel, the quality of the RNA extracted from the samples was assessed. Through proper application of DNase treatment, the DNA contamination in the isolated RNA was entirely removed. Utilising the Maxima First strand cDNA synthesis kit, the complementary DNA (cDNA) was created (ThermoScientific K1641).

### Quantification of IRF7 mRNA

**Figure 1.**
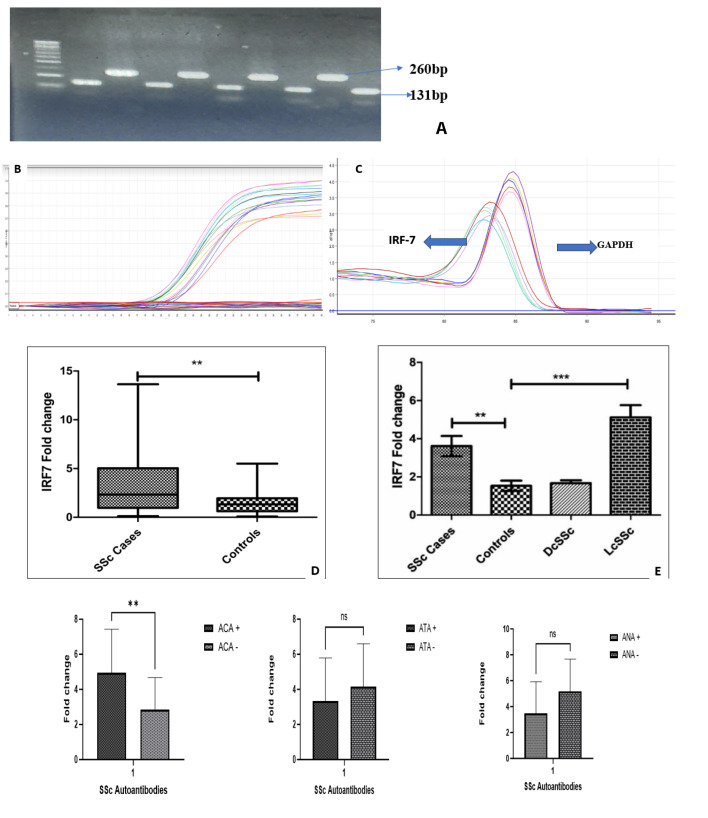
**(A)** cDNA amplicons of IRF7 and GAPDH gene. **(B & C)** Amplification plot and melt curve of IRF7 after qRT-PCR. **(D & E)** Fold change expression of IR-7 in SSc and its disease subsets. **(F)** mRNA expression analysis with SSc-specific autoantibody groups. **P=<0.01; ***P=<0.001, ns: non-significant.

The primer sequences for IRF7 (forward 5’-GCAAGTGCAAGGTGTACTG-3’ and reverse 5’-CACCAGCTCTTGGAAGAAGA-3’) and GAPDH (forward 5’- AGAAGGCTGGCTCATTTG-3’ and reverse 5’-AGGGGCCATCCACAGTTC-3’) were designed across exon-exon junctions (IDT software) to measure the IRF7 mRNA expression. The 20μl total volume of PCR reaction contained 10μl Master Mix, 2μl cDNA, 0.5μl of each primer, and 7μl of nuclease-free H_2_O. The above-mentioned reagents were pipetted in separate PCR tubes and placed in a Rotor-Gene Qiagen thermocycler. The thermocycler was performed at 95 °C for 3 min, followed by 40 cycles of 15 s at 95 °C and 1 min at 60 °C. Each set of reactions was repeated three times. The mean expression levels of the housekeeping gene GAPDH were utilised for standardisation. For the qPCR data analysis, the 2 ^– (ΔΔCt)^ method (Livak method) was utilised to calculate the relative expression of each target by normalising it to the internal GAPDH expression level. The successful RT-PCR amplification was assessed by resolving the amplicon on 3% agarose gel shown in **Figure 1A** and the amplification plot is shown in **Figure 1B& C**. To evaluate real-time PCR reactions for primer-dimer artifacts and to assure reaction specificity, post-amplification melting-curve analysis was done.

### Statistical Analysis

Data analysis was carried out using SPSS software (version 23.0; SPSS Inc., Chicago, IL, USA) and GraphPad Prism version 8 (GraphPad Software, La Jolla, CA, USA). The open EPI Statistical Software was used to determine the sample size. For categorical variables (such as sex and age) of the demographic data, the cases were compared using the Chi-square test. The normality of the data distribution for quantitative variables was determined using the Shapiro-Wilk test p-value, and histogram analysis. To assess the statistically significant difference between two or more groups, one-way ANOVA and the Student’s T-test were employed as parametric tests, respectively. The methylation data were dichotomised as 1 for the methylated allele only, 2 for the unmethylated both alleles, and 0 for the coexistence of the methylated and unmethylated alleles to facilitate statistical analysis using a contingency table. When p<0.05, statistical significance was taken into account.

## RESULTS

### Basic Characteristics of Study Subjects

At the entry, patients were profiled into dc SSc and lc SSc subgroups using the criteria proposed by LeRoy et al. 2013 and updated by the European League Against Rheumatism. All the patients follow American Rheumatological Criteria in our study. we examined a total of 60 subjects (40 patients with scleroderma and 20 normal controls) for expression and methylation analysis. Out of these, there were 90% female patients and 10% male patients. Most of the patients were found in rural areas (62.5%). On average, the patients were 35.02 years old, with a 9.40-year standard deviation. The mean duration of the disease was 4.97 years whereas the Raynaud’s Phenomenon average onset time was 5.47 years, with a 3.90 years standard deviation. When patients were stratified into subtypes, we found that out of the 40 patients with SSc, 26 had a lc SSc subset of SSc and 14 patients had a dc SSc subset. The mean age of patients in the lc SSc was 36.81 years with a standard deviation of 10.91, whereas the mean age of patients in the dc SSc was 36.02 years with a standard deviation of 8.20. Indicating the proper frequency matching, the mean age and gender distributions of cases and controls did not differ substantially. The basic characteristics of SSc subjects are outlined in **Table 1**.

**Table 1.** Basic characteristics of study subjects for mRNA expression and methylation analysis.

**Features**	**Cases (40)**	**Controls (20)**
Women	36	13
Age, y, mean±s.d	35.47±9.40	36.25±9.68
dcSSc	14	NA
lcSSc	26	NA
ANA	36	NA
ATA	25	NA
ACA	09	NA
Disease duration	4.97±2.51 years	NA
Onset age, y, mean±s.d	36.8±9.5 years	NA
Onset of Raynaud’s phenomenon	5.47±3.90 years	NA
Rural	25	12
Urban	15	08
Raynaud phenomenon (n)	35	NA
Digital ulcers	17	NA
Sclerodactyly	35	NA
Calcinosis	23	NA
Gangrene	10	NA
ILD	23	NA
PAH	20	NA
Puffy fingers	21	NA

### Autoantibodies and Disease Manifestations in SSc

Based on the clinical presentation of the SSc patients assessed, photosensitivity and skin thickening affect 95% of the patients. 92% of the patients had arthritis, telangiectasia, and sclerodactyly. During the initial examination, Raynaud’s phenomenon was found in about 95% of patients before the onset of the disease. Table 1 lists the key clinical characteristics of people with SSc. In our study, we observed that pulmonary arterial hypertension impacted 50% of patients, but pulmonary fibrosis or interstitial lung disease involved 57.5% of patients. After autoantibody testing, we found that over 90% of SSc patients tested positive for ANA, 22.5 % of patients were found to be ACA positive, and 62.5 % of patients were found to be ATA positive (**Table 1**). Although the difference was not statistically significant, we found that pulmonary fibrosis was more prevalent in the ATA group of patients whereas calcinosis, arthralgia, and telangiectasia were more prevalent in the ACA group.

### mRNA Expression Analysis in the Patient Group Compared with Healthy Controls

Compared to PBMCs from healthy individuals, it was found that the expression level of IRF7 mRNA was significantly elevated in SSc patients (fold change=3.61; p=0.003; **Figure 1D**) compared to controls. When patients were stratified into disease subsets it was found that the fold change of the lcSSc subset of SSc patients was significantly higher compared to the healthy control group (fold change=5.11; p<0.001) as shown in **Figure 1E**.

### mRNA Expression Analysis with SSc-Specific Autoantibody Groups

Further mRNA expression was correlated with disease-specific auto-antibodies, and results revealed that patients positive for ACA autoantibody had significantly higher IRF7 mRNA fold change compared to ACA-negative SSc patients (fold change=4.95; p=0.008; **Figure 1F**). No statistically significant associations were found for ANA or ATA autoantibody with IRF7 mRNA fold change in SSc patients (**Figure 1F**).

### IRF7 Promoter Methylation Analysis

**Figure 2.**
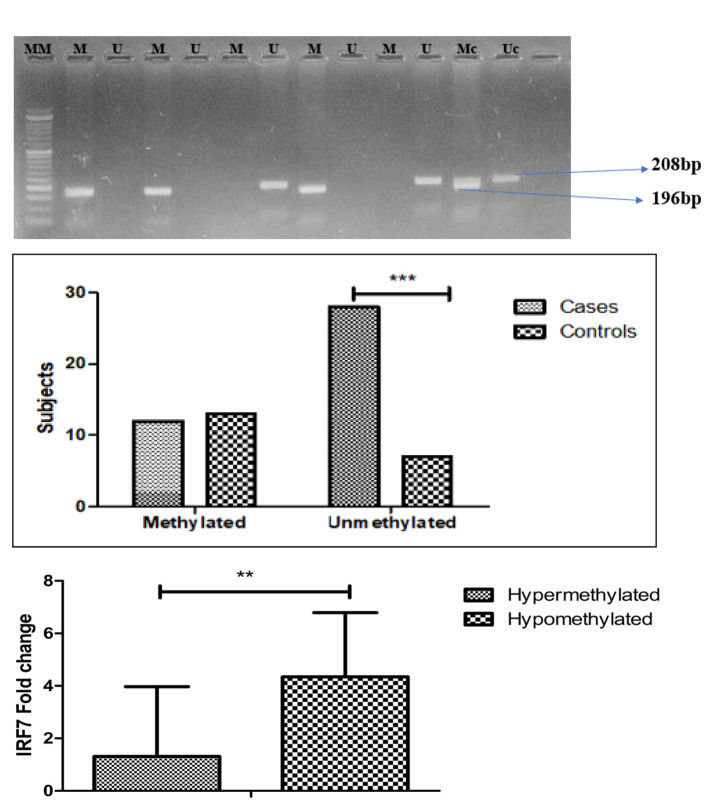
**(A)** Methylation-specific PCR results of IRF-7 promoter using specific primers. Lane MM: 50bp in DNA molecular weight marker; Lane M: Methylated band (196bp); Lane U: Unmethylated band (208bp); Lane Mc: Human Methylation (+) DNA Control; Lane Uc: Human Unmethylation (−) DNA Control. **(B)** Association of methylation status with cases and controls in systemic sclerosis. **(C)** Effect of IRF7 promoter methylation on its mRNA Expression. **P=<0.01; ***P=<0.001.

The methylation status of the IRF7 promoter was analysed in the same cohort of SSc patients (n=40) by methylation-specific PCR and compared with healthy controls (n=20) as shown in **Figure 2A**. In this study, most of the SSc cases had hypomethylated IRF7 promoters compared to controls (**Figure 2B**) and this difference in methylation pattern between the two groups was significant (p-value=0.001) as represented in **Table 2**. A significant increase in the IRF7 hypomethylation was observed in SSc cases as compared to healthy controls with a higher odds ratio (OR=4.33(1.38–13.56); p-value=0.009).

**Table 2.** IRF7 methylation in cases and controls and its impact on SSc.

**Methylation status**	**Cases**	**Controls**	**OR (95%CI)**	**p-value**
**Methylated**	12	13	**4.33(1.38–13.56)**	**0.009**
**Unmethylated**	28	07		

### Impact of Methylation on IRF7 mRNA Expression

We further aimed to analyse the impact of IRF7 promoter methylation on its mRNA expression and the results revealed that SSc cases (n=28) with unmethylated IRF7 promoter had increased mRNA expression of 4.34±2.45. In contrast, patients with the methylated promoter (n=12) depicted a decreased mean fold mRNA expression of 1.13±2.66. There was a significant difference in the expression of mRNA between the two groups. Therefore, we found that IRF7 promoter methylation status significantly influences IRF7 expression. **Figure 2C** depicts the effect of IRF7 promoter methylation on its mRNA expression.

## DISCUSSION

It is generally known that IFN-α has biological effects on both innate and acquired immunity and that an imbalance in IFN can lead to pathological autoimmunity, the destruction of immunological tolerance, and the consequent development of autoimmune diseases.^11^ IRFs are recognised to have a considerable impact on immune regulation. Initially identified as transcription factors, they control IFN and IFN-stimulated gene expressions. IRFs have biological functions that include controlling the IFN signalling pathway and the maturation, differentiation, and polarisation of immune cells.^12,13^ The transcriptional activation of type I IFN genes, which results in the production of proteins that are crucial immune system regulators, is significantly influenced by IRF7. Increased disease activity and specific autoantibody production have been linked to upregulated expression levels of type I IFN-inducible genes in SLE patients.^14,15^ A considerable percentage of SSc patients have been demonstrated to have elevated T1 IFN-regulated gene expression and activation.^16,17^ It has been reported that severe vascular symptoms, lung involvement, and antibody profile are all correlated with a greater IFN signature in SSc whole blood or plasma.^18,19^ In concordance with previous reports for other ethnic groups of patients with SSc,^20,21^ our study showed a significant increase in mRNA expression among Kashmiri patients confirming the relevance of IRF7 to SSc. Consistent with our study, Razaei et al. have also reported increased mRNA expression in SSc patients compared to normal healthy controls.^21^ The present study also reported a similar scenario whereby IRF7 was elevated in subjects with SSc, especially in the lcSSc subgroup, compared to healthy controls. IFNs cause B cells to produce autoantibodies, and when there is an excess of an antigen, this results in the development of immune complexes that contain DNA or RNA.^22^ In concordance with a previous study, our study also found a significant association between SSc-specific autoantibodies particularly ACA, and mRNA expression levels. Previous reports have established associations between the pathophysiology of SSc and all main epigenetic modifications, such as DNA methylation,^23,24^ histone modifications,^25,26^ and noncoding small (miRNA) expression.^27^ The autosomal genes of fibroblasts, immune cells, and endothelial cells have predominantly shown aberrant DNA methylation in SSc patients. Studies on SSc plasmacytoid dendritic cells (pDCs), specialised antigen-presenting cells that may quickly release a large amount of type I IFN upon activation, were motivated by the distinctive type I IFN signature discovered in SSc.^8^ Most of our SSc subjects had hypomethylation in the IRF7 promoter region, consequently leading to its increased mRNA expression. Additionally, the methylated and unmethylated promoter region of IRF7 showed significantly differing IRF7 mRNA expression levels in SSc patients. However, no significant association was observed between SSc-specific autoantibodies about DNA methylation percentile. Rezaei et al. also reported that DNA methylation plays an important role in developing SSc.^21,28^ Multiple immune-related disorders, including SLE and SSc, have been linked to type I IFN dysfunction.^7,14,27^ According to one study, CD4+ and CD8+ differentially methylated areas were substantially enriched in hypomethylation of genes related to the type I IFN signalling pathway.^23^ So, the epigenetic dysregulation of the type I IFN pathway in patients with systemic sclerosis suggests that type I IFN-associated gene overexpression and hypomethylation may play a crucial role in the aetiology of the disease. This is the first study to examine the role of IRF7 in the susceptibility of SSc and its clinical and serological subtypes in the Kashmiri SSc population. However, the main limitation of the study was the limited number of patients available, as patients with immune suppression were excluded from the study, and also the lack of disease controls in this study. Further study with a larger number of subjects would be helpful to substantiate the findings of this study.

## CONCLUSION

IRF7 promoter demethylation may cause IRF7 overexpression in PBMCs from SSc patients, and aberrant expression of IRF7 in SSc PBMCs may contribute to the pathogenesis of SSc.
